# Facilitating the Validation of Novel Protein Biomarkers for Dementia: An Optimal Workflow for the Development of Sandwich Immunoassays

**DOI:** 10.3389/fneur.2015.00202

**Published:** 2015-09-29

**Authors:** Marta del Campo, Wesley Jongbloed, Harry A. M. Twaalfhoven, Robert Veerhuis, Marinus A. Blankenstein, Charlotte E. Teunissen

**Affiliations:** ^1^Neurochemistry Laboratory, VU University Medical Center, Amsterdam, Netherlands; ^2^Department of Clinical Chemistry, VU University Medical Center, Amsterdam, Netherlands; ^3^Department of Psychiatry, VU University Medical Center, Amsterdam, Netherlands

**Keywords:** novel biomarkers, dementia, CSF, ELISA, workflow, guidelines, AD, FTD

## Abstract

Different neurodegenerative disorders, such as Alzheimer’s disease (AD) and frontotemporal dementia (FTD), lead to dementia syndromes. Dementia will pose a huge impact on society and thus it is essential to develop novel tools that are able to detect the earliest, most sensitive, discriminative, and dynamic biomarkers for each of the disorders. To date, the most common assays used in large-scale protein biomarker analysis are enzyme-linked immunosorbent assays (ELISA), such as the sandwich immunoassays, which are sensitive, practical, and easily implemented. However, due to the novelty of many candidate biomarkers identified during proteomics screening, such assays or the antibodies that specifically recognize the desired marker are often not available. The development and optimization of a new ELISA should be carried out with considerable caution since a poor planning can be costly, ineffective, time consuming, and it may lead to a misinterpretation of the findings. Previous guidelines described either the overall biomarker development in more general terms (i.e., the process from biomarker discovery to validation) or the specific steps of performing an ELISA procedure. However, a workflow describing and guiding the main issues in the development of a novel ELISA is missing. Here, we describe a specific and detailed workflow to develop and validate new ELISA for a successful and reliable validation of novel dementia biomarkers. The proposed workflow highlights the main issues in the development of an ELISA and covers several critical aspects, including production, screening, and selection of specific antibodies until optimal fine-tuning of the assay. Although these recommendations are designed to analyze novel biomarkers for dementia in cerebrospinal fluid, they are generally applicable for the development of immunoassays for biomarkers in other human body fluids or tissues. This workflow is designed to maximize the quality of the developed ELISA using a time- and cost-efficient strategy. This will facilitate the validation of the dementia biomarker candidates ultimately allowing accurate diagnostic conclusions.

## Introduction

Advancing age is the greatest risk factor of dementias, such as Alzheimer’s disease (AD), dementia with Lewy bodies (DLB), and frontotemporal dementia (FTD). As life span increases, dementia will impose a huge social and economic burden with more than 100 million of individuals predicted to suffer from dementia by 2050 worldwide (1). Up to now, there are no adequate treatment options to halt progression of the various types of neurodegenerative diseases leading to dementia. To be able to tailor treatment, it is important to determine the underlying pathological processes and the stage of progression of these processes at the individual level, before irreversible damage is done. Thus, there is a great interest in developing specific, sensitive, and practical tools to differentially diagnose and discriminate the different types of dementia in their earliest possible phase (i.e., AD, FTD, DLB, vascular dementia, etc.). Although the currently available cerebrospinal fluid (CSF) biomarkers for AD [i.e., amyloid β (Aβ), total Tau (t-Tau), and phosphorylated Tau (p-Tau) (2, 3)] have a high sensitivity and specificity for AD, there is still no test to effectively predict the development of AD in a pre-symptomatic stage (4). In addition, there are no biomarkers available for the diagnosis of other types of dementia, such as FTD or DLB (5). This can be partially attributed to the limited knowledge about the etiological factors underlying the neuropathology of the different disorders. Thus, there is an urgent need to unravel novel pathways and proteins in order to find new biomarkers reflecting the pathogenesis of the different dementia syndromes (i.e., AD, DLB, FTD), which will likely promote the development of novel alternative diagnostic and therapeutic strategies.

**Figure 1:**
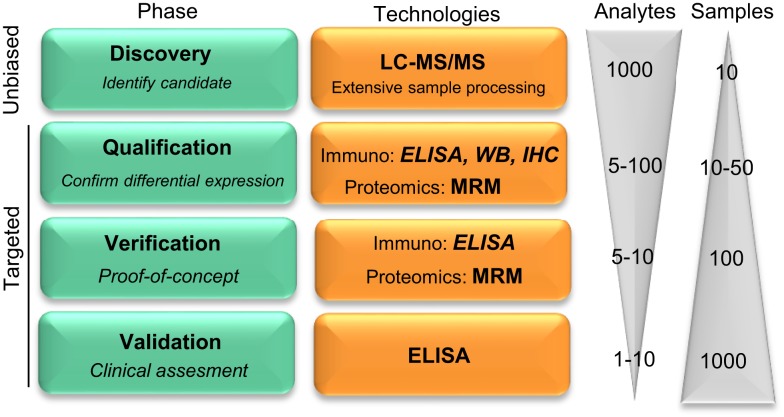
**Pipeline reflecting the development on novel protein biomarker candidates**. Biomarker development pipeline is divided into four main phases. Biomarker development starts with a low throughput screening of samples in the unbiased phase to a high throughput analysis in the latest clinical validation stage, where hundreds to thousands of samples are evaluated for the clinical assessment of the biomarker candidate. “Analytes” and “Samples” refer to the number of different protein targets or samples, respectively, that are evaluated in each phase. LC-MS/MS, liquid chromatography tandem mass spectrometry; MRM, multiple reaction monitoring; IHC, immunohistochemistry; WB, Western blotting. Figure adapted from Rifai et al. (11).

Global protein profiling by mass spectrometry (MS)-based proteomics has evolved as a new hypothesis-free (unbiased) avenue to optimally unravel new candidate protein biomarkers involved in different diseases, including neurodegenerative disorders (6). The sensitivity, speed, and the practicability of the different proteomics approaches has improved rapidly over the years (7, 8), leading to the discovery of an enormous number of biomarker candidates (9, 10). Most of the identified biomarker candidates have not yet been validated, which hampers their implementation in clinical practice (8). In order to facilitate the validation process, a coherent pipeline has been suggested for the development of novel biomarkers, which divides the overall process into four phases: discovery, qualification, verification, and validation (Figure 1) (11). Due to the high number of candidates identified in the discovery phase by unbiased proteomics [ranging between twenty and several hundred (10)], and the costs of assay development and validation, a prioritize selection of the discovered biomarker candidates should be performed (12) based on (i) the fold-change between control and disease cases, (ii) the possible relationship of the candidate with the pathological mechanisms, (iii) supporting literature, and/or (iv) the availability of the reagents to detect a specific target.

Noteworthy, unbiased-MS can only analyze a limited number of samples which, together with the extensive sample preparation required, leads to high false positive rate (13). Thus, the subsequent qualification phase serves to identify the potential false positive candidates and to confirm the differential abundance of the selected proteins using an alternative targeted methodology (11). During verification, prioritized markers are specifically analyzed in a larger cohort of samples. Among all the different technologies that are able to detect a specific protein in the qualification and verification phases, targeted proteomics [i.e., multiple reaction monitoring (MRM)] is a compelling option due to the higher accuracy and sensitivity compared to unbiased MS-approaches (10, 14). However, those techniques may not be readily available. Alternatively, antibody-based techniques [i.e., Western blotting, immunohistochemistry, enzyme-linked immunosorbent assays (ELISA)] can be used for qualification and verification. Due to the unbiased nature of the discovery phase, however, the specific reagents needed may not be commercially available, which will be the next critical issue during the validation phase. During validation, the reliability of the corresponding molecule as a biomarker is tested with the use of a highly specific assay that allows high throughput screening of samples.

**Figure 2:**
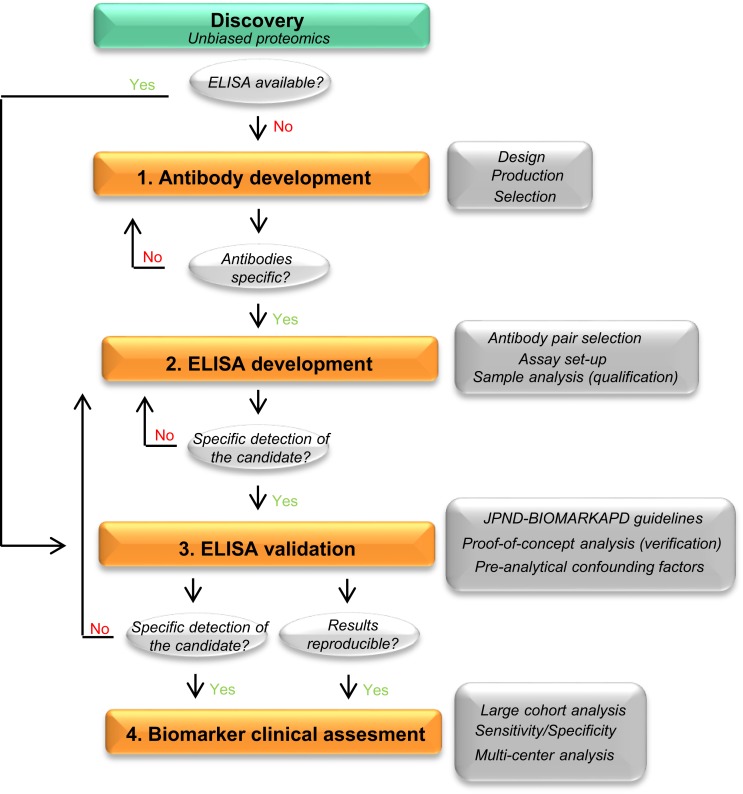
**Recommended workflow for the development of a novel ELISA**. Workflow to facilitate the development and analytical validation of assays for the validation of novel biomarker candidates. The process is divided into four different steps (orange rectangles). In each step, different analyses are performed (dark gray rectangles) and specific questions are addressed before moving into the next phase (light gray circles). When the different criteria in a specific phase cannot be reached, changes should be performed one phase back. If a specific ELISA is already available, it should undergo a validation process for the targeted matrix (step 3). JPND-BIOMARKAPD guidelines are published in this special issue by Andreasson and colleagues.

To date, the most accepted assay for biomarker validation is ELISA since it can measure numerous samples simultaneously with low variation (11). In addition, its use does not require highly qualified expertise or technology, allowing its implementation in every laboratory (14). Though different immunoassay formats are available, sandwich ELISA is the most common assay used in biomarker analysis due to its high specificity and sensitivity (15). In this format, the target protein will be detected using two different antibodies (capture and detection antibodies). For many of the candidate biomarkers, a commercially available assay will not exist and specific antibodies against the target of interest and/or the corresponding ELISA need to be developed. The development and optimization of an ELISA requires a careful design since a wide range of variables, ranging from the antibody specificity to the concentration and composition of the different reagents, can affect the final result and therefore the validity of the biomarker candidate. Thus, a careful design can reduce the development costs and ineffectiveness, and will probably lead to more accurate analytical outcomes. Previous guidelines described either the overall biomarker development in more general terms (i.e., the process from biomarker discovery to validation) (11) or how to perform the ELISA procedure itself (15), but not the main issues regarding the development of optimal ELISA for novel protein biomarker candidates in CSF. Here, we suggest a step-by-step workflow (Figure 2) to facilitate the development of new ELISA’s and the validation of novel biomarker candidates based on the literature available and our own best practice. In each step, different key issues need to be tested (Table 1). An estimated time-line for every step is also provided.

**Table 1:** **Critical issues of biomarker immunoassays**.

Workflow step	Key issue
1. Antibody production	Antibody design: optimal epitope selection Antibody production: polyclonal antibodies using peptides Internal control sample preparation Antibody selection: specificity tests
2. ELISA development	Optimal antibody pair selection (titration checkerboard) Assay set-up: concentrations, blocking buffers, etc. Biomarker candidate qualification
3. ELISA validation	JPND-BIOMARKAPD guidelines Pre-analytical confounding factors
4. Biomarker clinical assessment	Large cohort analysis: power analysis Biomarker sensitivity/specificity: AUC, ROC Reproducible results: multi-center studies

*CSF, cerebrospinal fluid; LOD, limit of detection, LOQ, limit of quantitation, AUC, area under the curve, ROC, receiver operating characteristic. JPND-BIOMARKAPD guidelines published in this special issue by Andreasson and colleagues*.

## Antibody Design, Production, and Selection

### Antibody design

**Figure 3:**
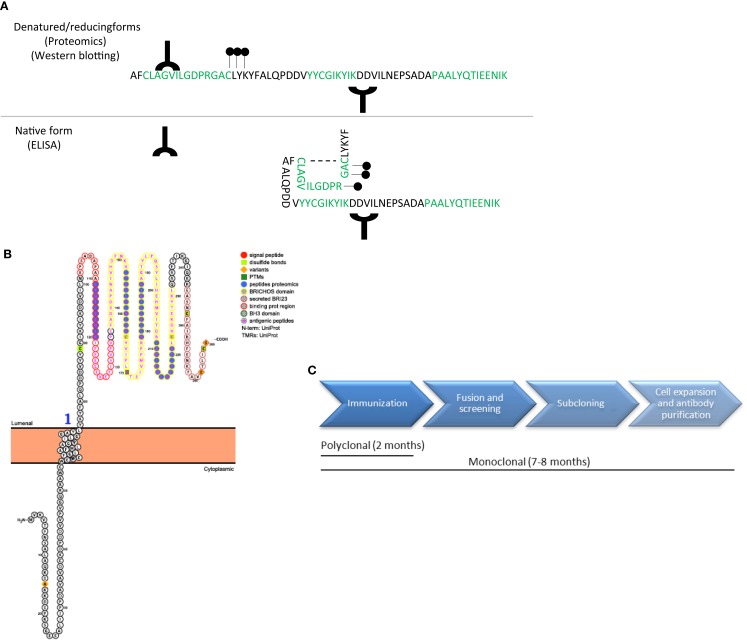
**Antibody design and production**. **(A)** Schematic representation of a protein amino acid sequence in denatured/reducing conditions (upper) or in its native form (bottom). Green amino acids represent the peptides recognized by unbiased proteomics in the discovery phase. Antibodies (Y symbol), glycol groups (black dots), and disulfide bonds (dotted line) are also represented. Specific epitopes detected in the unbiased proteomics approach might be available in denatured conditions but might be masked in native conditions. **(B)** Graphical representation of a transmembrane protein using Protter (20), in which all protein characteristics are included. **(C)** Time-line differences in the production of polyclonal and monoclonal antibodies.

The specificity and sensitivity of the antibody are the critical determinants defining the quality of an ELISA (16). It is essential that the antibodies used in the ELISA recognize the native protein or protein fragments in order to avoid sample processing and minimize variation of the final outcome. Noteworthy, the samples used to discover biomarker candidates are denatured, reduced, and trypsinized prior to analysis for the proteomics workup. Thus, the results of the unbiased approach provide information about unique peptides derived from proteins that are differently regulated between clinical groups. It is therefore important to have information about the protein characteristics, such as its 3D structure, hydrophobicity, post-translational modifications, and/or binding sites. For instance, an antibody developed against an epitope detected in the proteomics study that belongs to a highly hydrophobic or glycosylated part of the protein may not be suitable for ELISA since the corresponding epitope is masked under native conditions (17) (Figure 3A). Protein characteristics are accessible in different databases, such as the Universal Protein Resource (UniProt) or the protein data bank (PDB) (18, 19), but are also provided by companies specialized in antibody production. In addition, a novel online platform named Protter is very useful to get an overall representation of the target protein in which different annotations, including previous proteomics results or the known protein characteristics (binding and transmembrane domains, post-translational modifications, or cleavage sites) are presented (Figure 3B) (20).

### Immunogen selection

Based on the need to detect the native protein or protein fragments during the analysis to avoid sample processing, the optimal immunogen for antibody production should be the purified or recombinant full-length protein (21). However, the production and purification of full-length proteins is usually time consuming, costly and challenging from a technical perspective [i.e., aberrant protein folding, cells stress, solubility issues, etc. (22)]. In addition, the epitope recognized by the developed antibody might ultimately not be specific for the targeted native protein but rather to a general conformational state (17). Thus, it may be more effective to start antibody production using highly specific peptides. During peptide selection one should always consider: (i) the location of the peptide within the native protein and the post-translational modifications of the different epitopes within the protein to increase the chance that antibodies will detect the native protein and (ii) consider the peptide-ranges identified in the unbiased approach, since those are known to be differentially expressed in the clinical groups.

### Polyclonal vs. monoclonal antibodies

It is important to decide whether to use and produce polyclonal or monoclonal antibodies, which have their own advantages and disadvantages (23). Monoclonal antibodies are usually used in ELISA since, unlike polyclonals, they benefit from being derived from an indefinite source to produce exactly the same antibody (i.e., hybridoma cells), which significantly reduces batch-to-batch variation. Moreover, monoclonal antibodies are considered to be more specific than polyclonal since they recognize a single epitope. Nevertheless, if a small peptide is used for animal immunization (i.e., 15 amino acid), the different epitopes that the polyclonal antibodies can recognize are limited, equating the specificity between monoclonal and polyclonal antibodies. In addition, the time and thus the costs needed to produce monoclonal antibodies are considerably higher than those for polyclonal antibodies, which are therefore often chosen in early development stages. Rabbits are commonly used for polyclonal antibody production if there is no identified need for a specific animal species (i.e., remarkably large amounts of antibody needed) due to its easy handling, size, high titer, and high-affinity antiserum (24). Thus, we suggest starting with the production of polyclonal antibodies recognizing at least five different epitopes (one epitope per animal) within the protein. The affinity purification of the produced antibodies will remarkably increase the chances of obtaining specific signals. Large-scale production of monoclonal antibodies can start once the most reactive antibody to the targeted biomarker in the desired matrix is defined and the optimal antibody pairs for ELISA are identified.

Whenever available, it is recommended to select commercial antibodies based on a demonstrated high specificity (by, e.g., Western Blot of CSF or brain tissue) and described suitability for ELISA. In this respect, several initiatives that provide information about the antibodies available and their validation procedure are currently ongoing, such as the Antibody initiative of the Human Proteome Organization (25) or the Swedish Human Proteome Resource Program (26). Production of polyclonal antibodies may last at least 2 months (Figure 3C).

### Antibody reactivity and specificity

**Figure 4:**
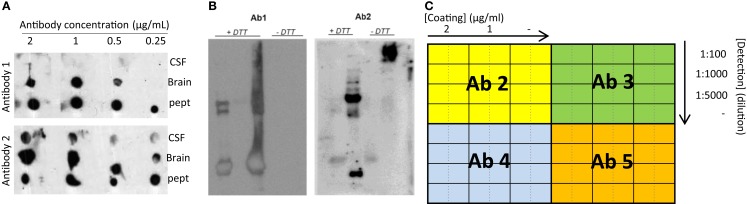
**Recommended experiments during antibody testing for an optimal selection of antibodies**. **(A)** Example of a dot blot against CSF, human brain, or antigenic peptide using two antibodies recognizing two different epitopes of the same protein at different concentrations. **(B)** Two different samples analyzed by Western blot under reducing (+DTT) and non-reducing (−DTT) conditions and detected by two antibodies (Ab1 and Ab2) detecting two different epitopes of the same protein. Only Ab 2 can detect the protein of interest in CSF in native conditions. **(C)** Schematic representation exemplifying a checkerboard titration to test different antibody pairs for ELISA development. In this set-up, one antibody (Ab1) is used as capture antibody in three different concentrations (2 and 1 μg/mL and no capture antibody (−)). Four different antibodies are tested as detection antibodies (Ab 2–5) in four different dilutions (1:100, 1:1000, 1:5000, and no detection antibody (−)). A fixed concentration of the standard protein/peptide is used in every well (i.e., 0.5 μg/mL). The best combination will be the one giving the highest signal using the lowest amount of antibody (higher dilution) and with the lowest background (signal when no antibody is used).

The specificity and reactivity of the different affinity-purified antibodies in different matrices (i.e., immunogen, CSF, tissue) can be tested using simple techniques, such as dot blot and Western blot. Some antibodies might be already excluded when no reactivity is observed (Figure 4A). In order to optimally compare the data between the different experiments, it is recommended to define and select a specific set of samples to be used continuously as internal controls (positive control sample) (27). For instance, individual CSF samples can be pooled into the different clinical groups (i.e., controls and AD) and aliquoted in order to have a large number of the same sample available. Pre-analytical variables (i.e., freeze/thaw cycles, storage temperature) affect the measurements of the CSF biomarkers (28–30) and thus the final outcome of the analyses. It is therefore important to follow specific guidelines for storage and handling of the CSF samples used in order to minimize the effect of possible pre-analytical bias already in this stage of development (31, 32). Since CSF is likely reflecting the biochemical alterations ongoing in the brain (33), it is conceivable to find changes of the identified proteins in brain tissue as well. Thus, when available, it is recommended to include also post-mortem brain tissue homogenates as it usually shows highly reactive bands. Noteworthy, our experience is that the height of the specific bands identified in brain tissue homogenates on Western blot is not identical to those in the CSF.

Testing antibody specificity in human samples can be challenging due to the lack of “pure” positive and negative controls (i.e., human samples lacking/overexpressing the target protein). Different types of reagents can be used to define specificity such as the recombinant full-protein, cell lysates, and/or animal tissue in which the target protein is overexpressed and/or downregulated (21). However, final conclusions for the specificity in human CSF or post-mortem tissue based only on reactivity observed in cells lysates or animal tissue must be drawn with due caution since this reactivity may not accurately represent the physiological form of the protein present in humans (21).

Antibody pre-adsorption with the antigenic peptide is also an easy and cost-effective alternative to test antibody specificity. If the signal obtained by Western blot using the antibody against the target matrix (e.g., CSF) is specific, it should be abrogated or remarkably reduced when the antibody is blocked with the antigenic peptide and be unaffected if similar but not identical peptides are used (34, 35). Unmodified reactivity after antibody pre-adsorption is non-specific and may derive from secondary antibody interactions or by contamination with other antibodies in the antibody solution (i.e., when the antibody has not been optimally affinity-purified). The reduced reactivity after antibody pre-adsorption does however not provide direct evidence of the specificity of the antibody, since the binding of the antibody to non-target proteins will be also inhibited. Thus, while persistent reactivity after pre-adsorption will indicate that the antibody is bad, reduced reactivity does not guarantee that the antibody is good (21, 36). Nonetheless, the binding to non-target proteins is unlikely to happen when antibodies have been produced against a unique sequence for the target protein. Further indirect evidence of antibody specificity comes from the comparison between the different antibodies, which should give a similar reactivity pattern (35). In addition, homologs of the target protein may exist. If the recombinant homologous protein/fragments or the antibodies against the homologous protein are available, it is recommended not only to compare the reactivities between the antibodies but also to test whether the newly developed antibodies targeting the biomarker candidate can recognize homologous proteins. Those analyses will help to rule out possible cross-reactivity.

Direct evidence of the specificity could be obtained via isolation of the proteins recognized by the antibody through immunopurification (IP) followed by mass-spectrometry analysis. Those analyses can however be costly and time consuming since larger amounts of human CSF samples are usually needed to obtain a meaningful signal and to prepare the negative controls (IP without antibody and/or with an irrelevant antibody), and protein isolation from the antibody–protein complex may result difficult due to a strong binding.

Based on the resources available, a combination of the different approaches should be applied to determine the specificity of the different antibodies, as it was previously done for the monoclonal antibody that was subsequently used for a specific ELISA against Aβ_40_ and not to other Aβ forms (37).

### Recognition of specific physiological protein forms

For a successful ELISA development, it is important to know the different possible conformational states of the target protein (monomers, dimers, aggregates) in the corresponding matrix (i.e., CSF) and thus samples should be analyzed under different denaturing and reducing conditions (Figure 4B). In addition to Western blotting, it is recommended to analyze samples via direct ELISA, in order to further test which antibodies are able to recognize both the recombinant protein/peptide and CSF in native conditions. At this stage, the type of ELISA plate should also be defined. The most common ELISA plate is the flat-bottomed 96-well polystyrene microplate, which allows the adsorption of the antibodies to the well plate by hydrophobic interactions (low and medium binding). Nevertheless, high binding microplates are also available, in which the surface is modified by radiation to increase the binding strength between the antibodies and the plate (38).

Antibodies with proven specificity and ability to recognize the native protein in the human samples are the optimal ones for further immunoassay development. If no optimal antibodies are found, new antibodies detecting different epitopes should be produced. Usually, when good antibodies are produced, this phase will last approximately 3 months of one full-time equivalent, though it will also depend on the number of antibodies as well as the availability of all the reagents, samples, and expertise needed.

## ELISA Development

### Antibody pair selection

A prerequisite for a good sandwich ELISA is that the two different antibodies (capture and detection antibodies) optimally match. Thus, the best antibody pairs able to detect the target CSF biomarker are identified by screening every possible combination. In order to avoid false positive measurements due to, e.g., direct reactivity between the antibodies, the optimal concentration of capture and detection antibodies for each combination has to be established. This can be done performing a checkerboard titration using the recombinant protein fragments/peptides (or full protein if available) at one fixed concentration (i.e., 0.5 μg/mL) as a standard sample/calibrator (Figure 4C). As a starting point, a concentration up to 2 and 10 μg/mL for capture and detection antibodies, respectively, can be tested. During the subsequent steps, it is recommended to always include the standard calibrators and the pooled positive control CSF samples previously prepared (27).

Once the optimal antibody concentrations are established for each combination, both the standard sample and the CSF pools should be measured in serial dilutions to define which antibody pairs are able to detect the target CSF protein and the corresponding standard in a dose–response manner. Dose–response reactivity gives a good indication that the antibody pairs are detecting the corresponding protein. Sample dilution experiments will unravel the standard curve range as well as the optimal dilution factor of the CSF. If dose–response reactivity is not acquired, this may indicate that the reactivity observed is non-specific. The source of the non-specific signal should be identified, which may arise among other possibilities from the detection system used (i.e., secondary antibodies) or an inadequate blocking buffer (see below). If the source of the non-specific signal is not identified and mitigated, the corresponding antibody pairs should not be used for further assay development.

Once results are successful, i.e., at least one or two positive antibody pairs are present, production of monoclonal antibodies recognizing the same epitopes can be considered. It is expected that the produced monoclonal antibodies will behave similar to the corresponding polyclonal due to the limited epitopes that were used for immunization of the latter. Depending on the number of antibodies to be tested and whether non-specific signal is detected, this phase may last from 3 to 10 months approximately.

### Assay set-up and fine-tuning

The different conditions and reagents used (i.e., incubation times, blocking buffers, assay diluent, secondary antibodies) can also play a critical role in the development of an ELISA (38). Blocking buffers are used to cover the unoccupied hydrophobic spaces of the ELISA plate wells once capture antibodies have been coated, reducing subsequent non-specific binding of the sample/reagents to the well. Different types of proteins are commonly used as a blocking agents, such as bovine serum albumin, non-fat dry milk, casein, normal serum, or fish gelatin, which can be diluted at different concentrations (ranging from 1 to 5%) in either phosphate- or tris-buffer saline (PBS or TBS). The different types of buffers and different protein concentrations should be tested since very low amount of blocking agent can lead to high background while excessive concentration may mask the binding epitope of the antibody. PBS can reduce signal of anti-phospho-epitope-specific antibodies, and in that case, TBS will be the first choice and should likewise be used for sample diluent. Non-ionic detergents can be added to the sample dilution buffer, such as Tween20 that disrupts low affinity protein–protein interactions and increases contact of the H_2_O-component of the buffer to the surface. However, when background is high, it is recommended to add the protein used for blocking to the sample diluent, though at a lower concentration. This detergent buffer is also used during the washing steps between the different incubations of an ELISA procedure, but usually without added proteins. Selecting the optimal diluent helps to keep the background low, this will lead to an increase of sensitivity of the assay and can reduce matrix effects.

In addition to the buffer requirements, one should select the detection system used to create a quantitative signal. Enzymes (i.e., Horseradish Peroxidase, alkaline phosphatase) are commonly used, which are attached to either the detection antibody, to a secondary antibody or streptavidin when biotinylated detection antibodies are used. The enzyme reaction will produce a specific color once the corresponding chromogenic substrate or fluorochrome has been added (i.e., 3,5,3′,5′-tetramethylbenzidine, p-nitrophenyl phosphate). The amount of signal generated within the linear range of the assay is proportional to the activity of enzyme present and thus, to the concentration of the target protein.

Once the different buffers and reagents have been established, it is recommended to re-test the optimal concentration of the coating and detection antibodies, since the improvements achieved with the different conditions may allow one to reduce the antibody concentration. Taking into account that only optimal antibody pairs are tested in this phase, it may take a maximum of 3 months to establish the best conditions leading to the highest signal/noise ratio for each of the antibody pairs. At this stage, a small number of individual patient samples should be tested. If available, it is recommended to use the same samples that were used during the discovery phase in order to replicate the proteomics findings (qualification).

## ELISA Validation

### Initial ELISA validation

Once an optimal assay has been developed or when it is commercially available, it is essential to test its analytical performance in the appropriate matrix (i.e., CSF) before assessing the clinical utility of the corresponding ELISA (28, 37, 39, 40). Several parameters need to be established such as precision, limits of detection, recovery, or parallelism among others. Validation of the assay will unravel whether the developed ELISA is accurate and robust in measuring the real levels of the candidate biomarker or if, on the contrary, the obtained values are influenced by other independent factors (i.e., pipetting errors, matrix effects, pre-analytical confounding factors). For example, the developed ELISA should have an optimal recovery, which is the ability of the assay to measure the specific candidate within the (complex) matrix (i.e., CSF) (41). During spike-recovery analysis, CSF samples with known concentration of the candidate biomarker are spiked with high, medium, low, and none amount of the calibrator. A bad recovery indicates that the different components of the matrix (i.e., CSF) affect the ability of the assay to measure the real concentration of the target molecule, which will affect the trueness of the results. Bad recoveries may be optimized by either using a different assay buffer mimicking better the matrix of interest or by further diluting the matrix of interest. ELISA validation will help to identify the different factors that compromise the reliability of the assay, which should be solved in order to draw accurate conclusions regarding the diagnostic performance of the biomarker candidate.

Previous guidelines have been published highlighting the parameters that should be stablished for the general validation of assays with different purposes (27, 41–45). This special issue in *Frontiers Neurology* contains a step-by-step and consensus standardized operating procedure (SOP) for a thorough ELISA validation for biomarkers for neurodegeneration (Andreasson et al.), developed by the members of the Joint Programming Neurodegenerative Disease (JPND) BIOMARKAPD (JPND-BIOMARKAPD), a consortium aiming to standardize the biomarker analysis for Alzheimer’s and Parkinson’s disease across Europe (46).

The fulfillment of the different parameters established by the JPND-BIOMARKAPD consortium as described by Andreasson and colleagues suggests that the assay is accurately measuring the candidate biomarker in CSF and thus a proof-of-concept analysis (verification) can be performed with a small cohort of individual samples (approximately 20 samples per clinical group). In case that some of the parameters are not fulfilled, it is recommended to re-analyze and test some of the incubation times, reagents, and concentrations established during assay development. Even if no changes in the concentration of the biomarker candidate are detected between the different clinical groups, it is worth to continue with a full-assay validation, since the assay might also be useful for other research purposes besides biomarker validation. However, full validation can only be performed on the final version of the assay. Noteworthy, when other matrices are used (i.e., post-mortem tissue, cell culture supernatants, cell lysates), an additional validation should always be performed to confirm the suitability of the assay for the corresponding matrix. The time frame for the completion of this phase typically lies between 2 and 8 months.

### Full ELISA validation

Once the new ELISA is fully developed, the novel assay should undergo an extensive validation for the targeted matrix in which other important parameters, including the reproducibility or the robustness of the assay, are tested as also indicated by Andreasson and colleagues in the current issue. The stability of the candidate biomarker under certain conditions should be also analyzed. Although the effect of pre-analytical variables have been likely minimized if the general guidelines for sample handling have been followed (31), some of the pre-analytical confounding factors should be specifically measured for the biomarker candidate to detect possible effects induced by different pre-analytical issues. Pre-analytical confounding factors include not only patient variables such as diurnal variation and fasting, but also processing factors such as the effect of freeze/thaw cycles and length of storage at different temperatures (28, 32, 47).

Since samples need to be prepared for pre-analytical variability testing (including storage over long time), this phase can take between 4 months and even a couple of years for long-term storage. Although the fulfillment of a complete ELISA validation ensures that the assay is suitable to measure the targeted molecule in the validated matrix, it is important to note that assay validation is a continuous process since reagents are continuously being renewed (i.e., quality control samples, standards, primary, and secondary antibodies). Thus, batch-to-batch variations should be always analyzed, tracked, and reported and, if needed, validation should be re-tested and an internal quality control program should be initiated. Nevertheless, since biomarker validation is a continuous process, current guidelines and workflows will have to be revised and updated regularly.

## Clinical Assessment of the Biomarker

The different processes followed until this point allow to successfully develop and to analytically validate an assay that can specifically and accurately measure the novel discovered CSF biomarker candidate. In this latest stage, the assay can be used for clinical validation of the biomarker for the intended purpose (i.e., diagnosis, prognosis, treatment efficiency). A considerably larger number of samples must be analyzed compared to the discovery phase and thus a power analysis should be performed in order to define the optimal group size. Specificity and sensitivity of the corresponding biomarker should be calculated and the ability of the biomarker to discriminate between control and disease can be assessed using the receiver operating characteristic (ROC) curve and the area under the curve (AUC), alone and/or in combination with currently used CSF biomarker tests (i.e., Aβ_42_, t-Tau, and p-Tau in AD) (48). According to international dementia biomarker criteria, a sensitivity and specificity of at least 85% is needed for a clinically useful biomarker (49). If a longitudinal study is performed, it might also be useful to assess the predictive value of the biomarkers that reflect the conversion from non-demented or mild cognitive impairment cases to the specific dementia with Cox proportional hazards models and Kaplan–Meier curves (50). When positive results are obtained, data should be independently replicated using larger cohorts, different populations and multi-center studies before its future possible implementation in routine analysis (51, 52).

## Concluding Remarks and Perspectives for Future Assay Implementation in Routine Analysis

There is a great need to develop specific, sensitive, and practical tools to differentially diagnose AD and related dementias in its earliest possible phase. The gold standard format for biomarker analysis is ELISA, which usually needs to be developed when a novel biomarker candidate is identified. In order to facilitate the development of a novel ELISA and ease the validation of the potential candidate CSF biomarkers, here we suggest a straightforward workflow for ELISA development, which we divided into four different steps (Figure 2). In each step, different key issues need to be tested (Table 1). When a commercial ELISA is available, the corresponding assay should be validated for its use in the corresponding matrix (i.e., CSF) (Figure 2, step 3). In the last steps, a clinical assessment of the biomarker candidate should be performed using the validated ELISA.

Once the optimal assay has been fully developed and validated, and the diagnostic utility of the corresponding biomarker has been solidly established, it will be necessary to initiate the phase that will ultimately lead to the implementation of the diagnostic assay in routine diagnosis, i.e., to establish an *in vitro* diagnostic (IVD) test. Such tests are preferably developed on an automated platform (53, 54), which will strongly reduce variation between centers allowing the establishment of cut-off values. In order to implement in clinical practice, several governmental requirements need to be fulfilled, which can range from the reproducibility and stability of the analytical platform to proof of added diagnostic value of the discovered biomarker. The exact set of rules that need to be complied to implement an IVD test depends on the regulatory institution of each region, which is, for example, the 510(k) premarketing clearance oversight by the food and drug administration in United States (55) or the IVD Directive 98/97/EC established by the European Commission (56).

Developing a successful ELISA for the validation of novel protein biomarker candidates starts by taking the right decisions during early stages of the development, for which we believe the workflow described in this paper will be a very useful aid.

## Conflict of Interest Statement

Dr. Teunissen serves on the advisory board of Fujirebio and Roche, received research consumables from Euroimmun, IBL, Fujirebio, Invitrogen, and Mesoscale Discovery. None of the other authors have any competing interest.
